# Complication, revision, and readmission rates following reverse total shoulder arthroplasty in wheelchair-dependent patients

**DOI:** 10.1016/j.jsea.2026.100003

**Published:** 2026-02-09

**Authors:** Henry M. Gass, Evan R. Simpson, Zachary T. Mills, Edward Quilligan, Hafiz F. Kassam

**Affiliations:** a Hoag Orthopedics, Irvine, CA, USA; b Kirk Kerkorian School of Medicine, University of Nevada Las Vegas, Las Vegas, NV, USA; c Hoag Orthopedic Institute, Irvine, CA, USA

**Keywords:** Reverse total shoulder arthroplasty, Wheelchair users, Shoulder arthroplasty outcomes, Periprosthetic fracture, Dislocation, Upper extremity weight-bearing

## Abstract

**Background:**

Wheelchair-dependent individuals place high mechanical demand on their upper extremities, relying on their shoulders as weight-bearing joints for propulsion and transfers. Although reverse total shoulder arthroplasty (rTSA) is effective for rotator cuff–deficient shoulders, outcomes in wheelchair users remain understudied. This study evaluated short-term complication and revision rates following rTSA in wheelchair-dependent patients using a large national claims database.

**Methods:**

A retrospective cohort study was performed using PearlDiver Mariner, identifying patients who underwent primary rTSA. Wheelchair dependence was defined by International Classification of Diseases-Ninth Revision, International Classification of Diseases-10th Revision, and Current Procedural Terminology codes. Wheelchair users were propensity score–matched 1:5 to controls by age, sex, Charlson-Deyo Comorbidity Index, and length of stay. The primary outcomes were 2-year revision rates and 90-day all-cause readmission and emergency department (ED) visits. Secondary analyses evaluated specific post-operative complications including dislocation, periprosthetic fracture, and infection.

**Results:**

Of 106,362 patients undergoing rTSA, 133 wheelchair users were matched to 665 controls. No significant difference was observed in 2-year revision rates (6.76% vs. 3.61%; *P* = .09). However, wheelchair users demonstrated higher 90-day rates of dislocation (4.51% vs. 1.65%; *P* = .048) and periprosthetic fracture (3.76% vs. 0.30%; *P* = .0019). All-cause 90-day ED utilization was also greater among wheelchair users (27.1% vs. 18.1%; *P* = .017).

**Conclusion:**

Wheelchair-dependent patients undergoing rTSA experience significantly higher early complication and ED visit rates, though 2-year revision rates appear similar. These findings highlight the unique mechanical and functional challenges faced by wheelchair users, underscoring the importance of specialized perioperative care and rehabilitation strategies in this high-demand population.

## Introduction

Wheelchair-dependent individuals place exceptional demands on their upper extremities. The glenohumeral joint in these patients serves not only for typical daily activities but also as primary weight-bearing joints during transfers or for propulsion. The cumulative mechanical load, frequent overuse, and altered biomechanics predispose this population to rotator cuff disorders, arthritis, pain, and loss of function, which can significantly diminish quality of life.^4^^,^^10^^,^^11^

Reverse total shoulder arthroplasty (rTSA) has emerged as a widely utilized surgical option for shoulder pathology in patients with rotator cuff deficiency, glenohumeral arthritis, fracture sequelae, and failed prior shoulder arthroplasty. In the general population, rTSA provides substantial improvements in pain, strength, and range of motion, with acceptable complication and revision rates, regardless of the indication.^8^^,^^15^^,^^24^ However, wheelchair users represent a special subgroup of patients for whom there is currently a limited amount of available literature in regard to outcomes such as revisions and complications, especially in regard to large database studies.

In recent years, several small case series and retrospective analyses have attempted to characterize rTSA outcomes in wheelchair-dependent populations. For example, Kemp and colleagues studied 19 wheelchair-dependent patients with lower-extremity impairment treated with rTSA and observed significant improvements in most functional scores, although with a roughly 25% complication rate, including issues like baseplate failure, dislocation, periprosthetic fracture, and high rates of glenoid notching.^12^ Similarly, Alentorn-Geli et al reported in a series of wheelchair users that rTSA produced significant pain relief and that 79% of patients had excellent or satisfactory outcomes, though a degree of morbidity was not uncommon in the first 2 to 5 years.^2^ Furthermore, in “weight-bearing shoulder” cohorts, which included wheelchair and crutch users, rTSA has been shown to improve motion, reduce pain, and enable resumption of many activities of daily living, albeit with a higher incidence of implant radiographic issues such as glenoid notching.^10^^,^^12^^,^^14^

Despite these promising findings, there are still persistent gaps in knowledge. The long-term durability of rTSA under repetitive weight-bearing loads, the precise rates of prosthetic complications in this population versus ambulatory patients, predictors of poorer functional recovery, optimal rehabilitation protocols, and patient satisfaction over extended follow-up remain incompletely defined. Another concern is that many of these studies are small case series, with relatively short follow-ups, and limited comparative data to a population of ambulatory patients.

Therefore, the aim of this study was to evaluate outcomes following rTSA in a larger subset of wheelchair users utilizing a large database. We assessed outcomes such as 2-year revision rate, 90-day all-cause readmission, and 90-day all-cause emergency department (ED) visits.

We hypothesize that while rTSA provides meaningful improvements in pain and function for wheelchair users, revision rates, complication rates, readmissions, and ED visits will exceed those in standard rTSA populations.

## Methods

### Study design

This retrospective cohort study was performed through the PearlDiver Mariner Patient Claims Database (PearlDiver Technologies, Colorado Springs, CO, USA) using the Mariner 170 dataset encompassing 170 million patients.^5^ The PearlDiver Patient Records Database contains deidentified patient-level information originating from insurance claims, which are aggregated and distributed commercially for research purposes. The dataset was queried from January 1, 2010, to April 30, 2023. All data are deidentified, and therefore this study is exempt from institutional review board approval. An ∗ indicates that an exact number is not known to avoid compromising deidentification of patient data.

### Patient selection

Patients were identified using International Classification of Diseases, Ninth Revision (ICD-9) codes, ICD, 10th Revision (ICD-10) codes, and Current Procedural Terminology codes. (Supplemental File 1) Patients were included if they underwent a primary rTSA and were longitudinally tracked from 1 month prior to the procedure to 2 years following the procedure. Wheelchair use was defined as wheelchair dependence (ICD-10-D-Z993 and ICD-9-D-V46.3) appearing on the same record as the procedure or within 1 month post-operatively. An additional code, Current Procedural Terminology-97542, was utilized as a verification step where a wheelchair was deemed medically necessary after the procedure to capture wheelchair users without a formal dependence diagnosis.^19^ Patients undergoing rTSA were stratified into 2 distinct cohorts: those who were wheelchair users and those who were not.

### Outcomes

The primary outcomes were revision within 2 years, 90-day all-cause readmission, and 90-day all-cause ED visits. Subgroup analysis was performed on the 90-day incidence of mechanical complications, dislocations, breakage, loosening, infection, other complications of device, implant, or graft, unspecified complications of internal joint prosthesis, periprosthetic fracture, other postprocedural musculoskeletal complication, and surgical operation with implant of artificial internal device. Ninety-day ED visits were also assessed for the incidence of the above reasons excluding surgical operation with implant of artificial internal device and including flail joint. All codes used for identification of outcomes can be found in Supplemental File 2.

### Statistical analysis

Descriptive statistics on cohort demographics were completed and reported as either mean and standard deviation or membership and percent. A Cox proportional hazards model was initially utilized to assess 2-year revision risk of wheelchair users with age, sex, Charlson-Deyo Comorbidity Index (CCI), and length of stay (LOS) controlled as covariates via interaction testing. The C-statistic was assessed for model performance with a score of 0.5 interpreted as random, 0.7 interpreted as a good model, and 1.0 as perfect prediction.^3^ This value indicated a moderate discriminatory effect, and thus a 5:1 propensity score match for age, sex, CCI, and LOS was performed using MatchIt in R within PearlDiver software (R Project for Statistical Computing, Vienna, Austria) to augment the analysis.^23^^,^^25^ The match utilized a nearest-neighbor algorithm with a 0.025 caliper to achieve balance between the control cohort and wheelchair user cohort. A Kaplan-Meier survivorship analysis of revision rate was performed on these cohorts and assessed with a log-rank test. All continuous variables were assessed with a Shapiro-Wilk test of normality. A Mann-Whitney U test was performed for all continuous outcomes. All binary outcomes were assessed using a 2-sided Pearson's chi-square test or, when expected counts were less than 5, a 2-sided Fisher's exact test. Statistical significance was defined as *P* < .05.

## Results

### Patient selection and characteristics

**Figure 1:**
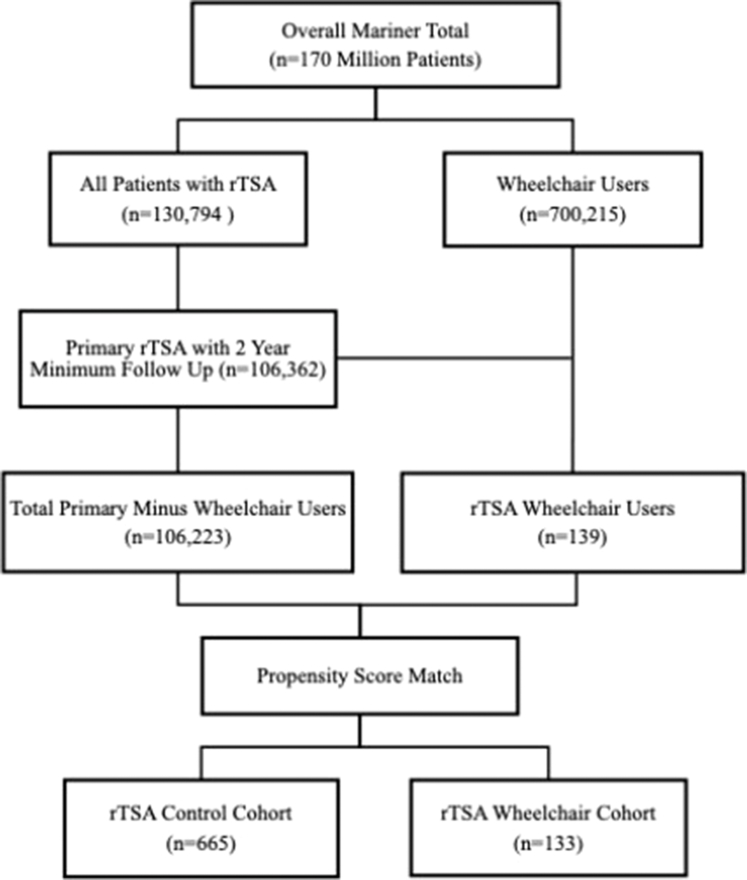
Flow diagram of patient identification and selection. *rTSA*, reverse total shoulder arthroplasty.

There were 106,362 patients who underwent primary rTSA. Propensity score matching resulted in 665 matched control patients to 133 wheelchair users (Fig. 1). The mean age was 68.9 ± 10.3 years in the wheelchair user cohort and 70.3 ± 8.3 years in the control cohort (*P* = .342). The CCI averaged 3.92 ± 2.59 and 4.26 ± 3.02 in the wheelchair and control cohorts, respectively (*P* = .447). Mean LOS was 3.02 ± 2.14 days in the wheelchair user cohort and 2.72 ± 1.92 days in the control cohort (*P* = .158). Male sex comprised 27.8% (n = 37) of the wheelchair user cohort and 23.6% (n = 157) of the control group (*P* = .356). A summary of patient characteristics can be found in Table I.

**Table I:** Baseline patient demographics of propensity-matched cohorts.

Characteristic	Control cohort	Wheelchair user cohort	*P* value
Total, n	665	133	-
Age (yr), mean ± SD	70.3 ± 8.3	68.9 ± 10.3	.342∗
Sex			.356†
Male, n (%)	157 (23.6%)	37 (27.8%)	-
Female, n (%)	508 (76.4%)	96 (72.2%)	-
CCI, mean ± SD	4.26 ± 3.02	3.92 ± 2.59	.447∗
Length of stay, mean ± SD	2.72 ± 1.92	3.02 ± 2.14	.158∗
Tobacco use, n (%)	§ (1.5%)	§ (2.26%)	.463‡
Payer, n (%)			.185†
Commercial	324 (48.7%)	68 (58.1%)	-
Government	§	§	-
Medicaid	29 (4.36%)	11 (8.27%)	-
Medicare	299 (45.0%)	50 (37.6%)	-
Unknown	§	§	-
Domains of CCI, n (%)			
Myocardial infarction	21 (3.15%)	§ (3.00%)	1^c^
Congestive heart failure	58 (8.72%)	13 (9.77%)	.697†
Peripheral vascular disease	30 (4.50%)	§ (3.00%)	.433†
Cerebrovascular disease	27 (4.06%)	§ (6.02%)	.315†
Dementia	§ (10.5%)	§ (4.51%)	.012‡
Chronic pulmonary disease	159 (23.9%)	41 (30.8%)	.093‡
Rheumatic disease	51 (7.7%)	§ (7.5%)	.952†
Peptic ulcer disease	§ (0.15%)	§ (0%)	1‡
Mild liver disease	15 (2.26%)	§ (1.5%)	.752‡
Diabetes without chronic complication	162 (24.4%)	19 (14.3%)	.011†
Diabetes with chronic complication	74 (11.1%)	12 (9.02%)	.475†
Hemiplegia or paraplegia	§ (0.45%)	18 (13.5%)	<.001
Renal disease	94 (14.1%)	18 (13.5%)	.855†
Any malignancy	19 (2.85%)	§ (1.5%)	.555‡
Moderate or severe liver disease	§ (0.3%)	§ (1.5%)	.132‡
Metastatic solid tumor	§ (1.05%)	§ (0.75%)	1‡
AIDS/HIV	§ (0.15%)	§ (0%)	1‡

*CCI*, Charlson-Deyo Comorbidity Index; *SD*, standard deviation.

∗ Mann–Whitney U test.

† Two-sided chi-square test.

‡ Two-sided Fisher's exact test.

§ Indicates data suppressed to protect deidentification.

### Two-year revision

The multivariate Cox proportional hazards model found increasing age reduced the hazard of revision (hazard ratio [HR] = 0.9561; 95% confidence interval [CI]: 0.95–0.96; *P* < .001), whereas greater CCI (HR = 1.05; 95% CI: 1.04–1.07; *P* < .001) and LOS (HR = 1.03; 95% CI: 1.02–1.06; *P* < .001) were associated with an increased hazard of revision. Male sex was associated with a significant increase in hazard compared to female sex (HR = 1.88; 95% CI: 1.73–2.04; *P* < .001). Assessment of the model indicated moderate discriminative performance, C-statistic = 0.651 (standard error = 0.005). Subsequent analysis was performed using propensity-matched cohorts to address variation unaccounted for in the model and ensure comparability between groups. Kaplan-Meier survivorship curves compared via a log-rank test demonstrated a nonsignificant difference in 2-year revision rate between wheelchair users (6.76%, n = ∗) and matched controls (3.61%, n = 24, *P* = .09).

### Ninety-day readmission and outcomes

Within 90 days post-operatively of primary rTSA, rates of dislocations and periprosthetic fractures were significantly higher in wheelchair users when compared to propensity-matched controls. Dislocations occurred in 4.51% (n = ∗) of wheelchair users and in 1.65% (n = 11) of controls (*P* = .048). Periprosthetic fractures occurred in 3.76% (n = ∗) of wheelchair users and in 0.30% (n = ∗) of controls (*P* = .0019). Complete event frequencies and nonsignificant 90-day outcomes are listed in Table II.

**Table II:** Ninety-day post-operative outcomes.

Outcome	Control cohort	Wheelchair user cohort	*P* value
Readmissions, n (%)	167 (25.11%)	39 (29.32%)	.311∗
Mechanical complications, n (%)	‡ (1.05%)	‡ (3.01%)	.094†
Dislocations, n (%)	11 (1.65%)	‡ (4.51%)	.048†
Breakage, n (%)	0	0	1†
Loosening, n (%)	‡ (0.301%)	‡ (1.50%)	.132†
Infection, n (%)	‡ (1.05%)	‡ (1.50%)	.650†
Other complications of device, implant, for graft, n (%)	‡ (1.05%)	0	.608†
Unspecified complications of internal joint prosthesis, n (%)	‡ (.301%)	0	1†
Periprosthetic fracture, n (%)	‡ (0.301%)	‡ (3.76%)	.0019†
Other postprocedural musculoskeletal complication, n (%)	‡ (0.45%)	0	1†
Surgical operation with implant of artificial internal device, n (%)	‡ (0.45%)	0	1†

∗ Two-sided chi-square test.

† Two-sided Fisher's exact test.

‡ Indicates data suppressed to protect deidentification.

### Ninety-day emergency department visits

Analysis of ED utilization within 90 days following a rTSA demonstrated increased all-cause 90-day ED visit rates among wheelchair users (27.1% [n = 36], *P* = .017) compared to controls (18.1% [n = 120], *P* = .017). Periprosthetic fractures accounted for 1.5% (n = ∗) of wheelchair user 90-day ED visits compared to 0% of the control cohort ([n = ∗], *P* = .028) while other ED visit causes showed no significant differences as detailed in Table III.

**Table III:** Ninety-day ED visits.

ED visit reason	Control cohort	Wheelchair user cohort	*P* value
All-cause visits, n (%)	120 (18.05%)	36 (27.07%)	.017∗
Mechanical complications, n (%)	0	0	1†
Dislocations, n (%)	‡ (0.150%)	‡ (0.75%)	.306†
Breakage, n (%)	0	0	1†
Loosening, n (%)	0	0	1†
Infection, n (%)	‡ (0.150%)	0	1†
Other complications of device, implant, for graft, n (%)	‡ (0.150%)	0	1†
Unspecified complications of internal joint prosthesis, n (%)	0	0	1†
Periprosthetic fracture, n (%)	0	‡ (1.5%)	.028†
Other postprocedural musculoskeletal complication, n (%)	0	0	1†
Flail joint, n (%)	0	0	1†

*ED*, emergency department.

∗ Two-sided chi-square test.

† Two-sided Fisher's exact test.

‡ Indicates data suppressed to protect deidentification.

## Discussion

This study represents one of the largest database analyses to date evaluating outcomes following rTSA in wheelchair-dependent patients. Utilizing a national administrative claims database, we identified higher complication rates, particularly dislocations, periprosthetic fractures, and ED utilization, among wheelchair users compared to matched ambulatory controls. However, there was no statistically significant difference in 2-year revision rates. These findings suggest that while rTSA remains a viable reconstructive option for shoulder pathology in wheelchair users, this population may be at greater risk of early post-operative complications that warrant special perioperative consideration.

Our results are consistent with and expand upon findings from prior small case series in the literature. Kemp et al reported on 19 wheelchair-dependent patients undergoing rTSA and found a 25% overall complication rate, including dislocation, baseplate failure, and periprosthetic fracture, despite significant improvements in pain and function.^12^ Similarly, Alentorn-Geli et al demonstrated satisfactory pain relief and functional improvement in 79% of their wheelchair cohort but noted a relatively high incidence of medical and prosthetic complications within five years.^2^^,^^19^ Levy and colleagues also reported favorable functional outcomes in “weight-bearing shoulders,” including both wheelchair and crutch users, though their study showed an incidence of glenoid notching in 20% of patients but no radiographic evidence of instability, subsidence, stress shielding, or loosening.^14^

Tate et al looked at the complication rates for rTSA in the PearlDiver database and found the overall complication rate in patients with osteoarthritis to be 3.8%.^21^ Our database findings corroborate these observations but at a much larger scale, supporting that wheelchair dependence is associated with acceptable prosthesis survival in the short term but an elevated early complication burden compared to ambulatory patients, particularly with regard to instability.

The mechanisms underlying these elevated complication rates are likely multifactorial. Wheelchair propulsion, transfers, and pressure-relief maneuvers place repetitive axial and shear loads on the glenohumeral joint, increasing stress on the prosthetic components and soft.^4^ The use of the upper extremities as weight-bearing limbs creates high cyclical loads that can predispose to instability, component loosening, or periprosthetic fractures.^22^ This was particularly more prevalent in the wheelchair-dependent cohort. Additionally, wheelchair users can present with significant comorbidities, such as spinal cord injury, stroke, or lower-extremity amputation, each of which may contribute to frailty, altered proprioception, and impaired rehabilitation potential.^2^^,^^12^

Interestingly, our study did not demonstrate a statistically significant increase in 2-year revision rates among wheelchair users despite these increased early complications. This may reflect the limited follow-up interval, given that mechanical failures such as loosening or baseplate migration often manifest beyond two years post-operatively.^9^ Alternatively, some complications in wheelchair users may be managed nonoperatively due to medical complexity or patient preference, which could underestimate true prosthetic failure rates in administrative datasets. Future research with longer follow-up and detailed clinical data is warranted to better characterize implant survivorship in this population.

Another noteworthy finding of this study is the significantly higher rate of ED utilization within 90 days post-operatively among wheelchair users. While increased medical comorbidities in wheelchair users likely contribute to this trend, it may also reflect the challenges of recovery and self-care after upper extremity surgery in patients who rely on their shoulders for mobility.^13^^,^^24^ Early post-operative immobility, transfer limitations, and dependence on caregivers can create additional risks for falls, wound issues, or contralateral shoulder strain. Enhanced post-operative support and tailored rehabilitation protocols, potentially including temporary powered mobility devices, may mitigate some of these early risks.

From a surgical perspective, optimizing implant stability and soft tissue tensioning may be particularly critical for wheelchair users who are expected to repetitively load their shoulders. Some authors have suggested consideration of constrained liners or augmented glenoid components to decrease instability.^16^ Additionally, specific design and implant positioning factors are known to improve outcomes in rTSA in general and would certainly only be amplified in the wheelchair population.^6^^,^^7^ However, further biomechanical and clinical data are needed to define best practices for component positioning and fixation in this unique population.

In addition to patient-related biomechanical demands, implant-specific factors may substantially influence instability and complication profile following rTSA in wheelchair-dependent patients. Larger glenosphere diameters increase jump distance and improve intrinsic construct stability, requiring more force to dislocate than smaller, neutral offset glenospheres.^17^ Therefore, implant design may play a significant role in this population and may be particularly beneficial in individuals exposed to repetitive axial loading during transfers and propulsion. Registry-level data from the Australian Orthopaedic Association National Joint Replacement Registry demonstrated a significantly lower risk of revision with increasing glenosphere diameter, supporting the use of larger glenospheres in high-demand populations.^18^ Similarly, implant lateralization strategies have been associated with improved deltoid tensioning, reduced impingement, and enhanced stability, although excessive lateralization may increase acromial and scapular spine stress, highlighting the need for balanced construct selection. From a clinical perspective, Frankle's classification of instability following rTSA, focusing on loss of compression, loss of containment, and impingement emphasizes the multifactorial nature of post-operative instability. Component positioning, soft tissue tensioning, and deltoid function provide a useful framework to guide implant selection and surgical technique in patients at elevated risk.^1^ Lastly, neck shaft angle has been shown to play a significant role in horizontal stability of rTSA in biomechanical studies, with higher neck shaft angle of 155° requiring a higher dislocation force than lower neck shaft angles such as 135 or 145°.^20^ A higher dislocation force could be mechanically advantageous in arm ambulators who may put an increase force through their implants.

Furthermore, pre-operative factors such as the degree of rotator cuff deficiency, residual posterior cuff function, and subscapularis integrity may influence post-operative stability and should be considered when planning implant configuration and soft tissue management in wheelchair users. Collectively, these considerations suggest that thoughtful implant selection and attention to soft tissue balance may represent modifiable strategies to mitigate complication risk in this challenging cohort.

This study has several limitations inherent to large database analyses. First, reliance on administrative coding introduces the potential for misclassification of wheelchair use and post-operative complications. We attempted to mitigate this by incorporating both diagnosis and procedural codes for wheelchair dependence. Second, the PearlDiver database does not include detailed clinical or radiographic data such as functional scores, implant type or design, or surgical technique, precluding analysis of range of motion, pain relief, or radiographic outcomes. Additionally, follow-up was limited to two years; longer-term implant survival remains unknown. Finally, while propensity score matching improved comparability between cohorts, unmeasured confounding factors such as pre-operative shoulder function, bone quality, or rehabilitation intensity may still have influenced outcomes.

## Conclusion

In this large national database analysis, wheelchair-dependent patients undergoing rTSA experienced significantly higher short-term complication and ED visit rates compared to ambulatory controls, though 2-year revision rates were not significantly different. These findings emphasize the need for careful pre-operative counseling, meticulous surgical technique, and post-operative support tailored to the unique biomechanical and functional demands of wheelchair users. Further prospective, long-term studies are warranted to clarify implant durability, optimal rehabilitation strategies, and patient-reported outcomes in this challenging population.

## Disclaimer:

Funding: No funding was disclosed by the authors.

Conflicts of interest: The other authors, their immediate families, and any research foundations with which they are affiliated have not received any financial payments or other benefits from any commercial entity related to the subject of this article.

## References

[1] Abdelfattah A., Otto R.J., Simon P., Christmas K.N., Tanner G., LaMartina J. (2018). Classification of instability after reverse shoulder arthroplasty guides surgical management and outcomes. J Shoulder Elbow Surg.

[2] Alentorn-Geli E., Wanderman N.R., Assenmacher A.T., Sánchez-Sotelo J., Cofield R.H., Sperling J.W. (2018). Reverse shoulder arthroplasty in weight-bearing shoulders of wheelchair-dependent patients: outcomes and complications at 2 to 5 years. PM R.

[3] Baessler A.M., Brolin T.J., Azar F.M., Sen S., Chang M., Falkner D. (2021). Development and validation of a predictive model for outcomes in shoulder arthroplasty: a multicenter analysis of nearly 2000 patients. J Shoulder Elbow Surg.

[4] Bayley J.C., Cochran T.P., Sledge C.B. (1987). The weight-bearing shoulder. The impingement syndrome in paraplegics. J Bone Joint Surg Am.

[5] Bolognesi M.P., Habermann E.B. (2022). Commercial claims data sources: pearldiver and individual payer databases. J Bone Joint Surg Am.

[6] Brune D., George S.Z., Edwards R.R., Moroder P., Scheibel M., Lazaridou A. (2024). Which patient level factors predict persistent pain after reverse total shoulder arthroplasty?. J Orthop Surg Res.

[7] Callegari J., Haidamous G., Lädermann A., Phillips C., Tracy S., Denard P. (2021). Factors influencing appropriate implant selection and position in reverse total shoulder arthroplasty. Orthop Clin North Am.

[8] Crutsen J.R.W., Lambers Heerspink F.O., van Leent E.A.P., Janssen E.R.C. (2024). Predictive factors for postoperative outcomes after reverse shoulder arthroplasty: a systematic review. BMC Musculoskelet Disord.

[9] Holcomb J.O., Cuff D., Petersen S.A., Pupello D.R., Frankle M.A. (2009). Revision reverse shoulder arthroplasty for glenoid baseplate failure after primary reverse shoulder arthroplasty. J Shoulder Elbow Surg.

[10] Jordan R.W., Sloan R., Saithna A. (2018). Should we avoid shoulder surgery in wheelchair users? A systematic review of outcomes and complications. Orthop Traumatol Surg Res.

[11] Jung H.J., Sim G.B., Jeon I.H., Kekatpure A.L., Sun J.H., Chun J.M. (2015). Reconstruction of rotator cuff tears in wheelchair-bound paraplegic patients. J Shoulder Elbow Surg.

[12] Kemp A.L., King J.J., Farmer K.W., Wright T.W. (2016). Reverse total shoulder arthroplasty in wheelchair-dependent patients. J Shoulder Elbow Surg.

[13] LaBerge N.B., Detterbeck A., Nooijen C.F.J. (2023). Comorbidities and medical complexities of mobility device users: a retrospective study. Disabil Rehabil Assistive Technol.

[14] Levy O., Arealis G., Tsvieli O., Consigliere P., Lubovsky O. (2024). Reverse total shoulder replacement for patients with “weight-bearing” shoulders. Clin Shoulder Elb.

[15] Lindbloom B.J., Christmas K.N., Downes K., Simon P., McLendon P.B., Hess A.V. (2019). Is there a relationship between preoperative diagnosis and clinical outcomes in reverse shoulder arthroplasty? An experience in 699 shoulders. J Shoulder Elbow Surg.

[16] Nakazawa K., Manaka T., Minoda Y., Hirakawa Y., Ito Y., Iio R. (2024). Impact of constrained humeral liner on impingement-free range of motion and impingement type in reverse shoulder arthroplasty using a computer simulation. J Shoulder Elbow Surg.

[17] Nguyen N., Huang Y., Eckers F., Ek E.T., Ernstbrunner L., Ackland D.C. (2025). The influence of glenosphere size and glenoid-sided offset on shoulder stability following reverse total shoulder arthroplasty using the Zimmer Trabecular Metal Reverse plus shoulder system. J Shoulder Elbow Surg.

[18] Page R., Beazley J., Graves S., Rainbird S., Peng Y. (2022). Effect of glenosphere size on reverse shoulder arthroplasty revision rate: an analysis from the Australian Orthopaedic Association National Joint Replacement Registry (AOANJRR). J Shoulder Elbow Surg.

[19] Prabhu K., Nasr A.J., Kasitinon D., Cabrera A., Lin Y.S. (2023). Perioperative outcomes, comorbidities, and complications following total shoulder arthroplasty in wheelchair users: a retrospective cohort analysis of a nationwide database. J Clin Med.

[20] Shimada Y., Ochiai N., Hashimoto E., Hirosawa N., Nojima D., Kajiwara D. (2022). Effect of the neck-shaft angle on stability in the onlay type of reverse shoulder arthroplasty: a cadaveric study. Semin Arthroplasty.

[21] Tate J.P., Reinhart N.M., Troutman T.M., Sherman W.F., O’Brien M.J. (2025). Reoperation roulette: unveiling diagnosis-specific complication rates in anatomic and reverse total shoulder arthroplasty. Semin Arthroplasty.

[22] Valiquette A.M., Graf A.R., Mickschl D.J., Zganjar A.J., Grindel S.I. (2022). Rotator cuff repair in upper extremity ambulators: an assessment of longitudinal outcomes. JSES Int.

[23] Wang Y., Griffin J., Werner B. (2023). Shoulder arthroplasty in patients with multiple sclerosis: complications stratified by arthroplasty type. Semin Arthroplasty.

[24] Werner B.C., Wong A.C., Mahony G.T., Craig E.V., Dines D.M., Warren R.F. (2016). Causes of poor postoperative improvement after reverse total shoulder arthroplasty. J Shoulder Elbow Surg.

[25] Zhang Z. (2017). Propensity score method: a non-parametric technique to reduce model dependence. Ann Transl Med.

