# Supplementary material for: Complication, revision, and readmission rates following reverse total shoulder arthroplasty in wheelchair-dependent patients
Source: J Shoulder Elb Arthroplast. 2026 Feb 9;10(1-2):100003. doi: 10.1016/j.jsea.2026.100003 (PMC13012886; doi:10.1016/j.jsea.2026.100003)
Supplement: Supplemental File 1 [file mmc1.docx]

**Supplemental File 1.** ICD 9, ICD 10, and CPT codes for Identification of Patients and Relevant Demographics

**Reverse Shoulder Arthroplasty:**

ICD-9-P-8188 - Reverse Total Shoulder Replacement

ICD-10-P-0RRJ00Z - Replacement of Right Shoulder Joint with Reverse Ball and Socket Synthetic Substitute, Open Approach

ICD-10-P-0RRK00Z - Replacement of Left Shoulder Joint with Reverse Ball and Socket Synthetic Substitute, Open Approach

**Wheelchair Use Identification:**

ICD-9-D-V46.3: Wheelchair Dependence

ICD-10-D-Z99.3: Dependence on a wheelchair

CPT-97542 - Management of a patient using a wheelchair including assessment (pos- tural/positioning needs), fitting (pressure relief)

**Tobacco Use:**

ICD-9-D-V15.82 - Personal history of tobacco use

ICD-10-D-Z72.0 - Tobacco use

**Charlson Comorbidity Index Domains:**

Myocardial Infarction - ICD-9-D-410.x, ICD-9-D-412.x, ICD-10-D-I21.x, ICD-10-D-I22.x, ICD-10-D-I25.2

Congestive Heart Failure - ICD-9-D-398.91, ICD-9-D-402.01, ICD-9-D-402.11, ICD-9-D-402.91, ICD-9-D-404.01, ICD-9-D-404.03, ICD-9-D-404.11, ICD-9-D-404.13, ICD-9-D-404.91, ICD-9-D-404.93, ICD-9-D-425.4-425.9, ICD-9-D-428.x, ICD-10-D-I09.9, ICD-10-D-I11.0, ICD-10-D-I13.0, ICD-10-D-I13.2, ICD-10-D-I25.5, ICD-10-D-I42.0, ICD-10-D-I42.5-I42.9, ICD-10-D-I43.x, ICD-10-D-I50.x, ICD-10-D-P29.0

Peripheral Vascular Disease - ICD-9-D-093.0, ICD-9-D-437.3, ICD-9-D-440.x, ICD-9-D-441.x, ICD-9-D-443.1-443.9, ICD-9-D-447.1, ICD-9-D-557.1, ICD-9-D-557.9, ICD-9-D-V43.4, ICD-10-D-I70.x, ICD-10-D-I71.x, ICD-10-D-I73.1, ICD-10-D-I73.8, ICD-10-D-I73.9, ICD-10-D-I77.1, ICD-10-D-I79.0, ICD-10-D-I79.2, ICD-10-D-K55.1, ICD-10-D-K55.8, ICD-10-D-K55.9, ICD-10-D-Z95.8, ICD-10-D-Z95.9

Cerebrovascular Disease - ICD-9-D-362.34, ICD-9-D-430.x-438.x, ICD-10-D-G45.x, ICD-10-D-G46.x, ICD-10-D-H34.0, ICD-10-D-I60.x-I69.x

Dementia - ICD-9-D-362.34, ICD-9-D-430.x-438.x, ICD-10-D-G45.x, ICD-10-D-G46.x, ICD-10-D-H34.0, ICD-10-D-I60.x-I69.x

Chronic Pulmonary Disease - ICD-9-D-416.8, ICD-9-D-416.9, ICD-9-D-490.x-505.x, ICD-9-D-506.4, ICD-9-D-508.1, ICD-9-D-508.8, , ICD-10-D-I27.9, ICD-10-D-J40.x-J47.x, ICD-10-D-J60.x-J67.x, ICD-10-D-J68.4, ICD-10-D-J70.1, ICD-10-D-J70.3

Rheumatic Disease - ICD-9-D-446.5, ICD-9-D-710.0-710.4, ICD-9-D-714.0-714.2, ICD-9-D-714.8, ICD-9-D-725.x, ICD-10-D-M05.x, ICD-10-D-M06.x, ICD-10-D-M31.5, ICD-10-D-M32.x-M34.x, ICD-10-D-M35.1, ICD-10-D-M35.3, ICD-10-D-M36.0

Peptic Ulcer Disease – ICD-9-D-531.x-534.x, ICD-10-D-K25.x-K28.x

Mild Liver Disease - ICD-9-D-070.22, ICD-9-D-070.23, ICD-9-D-070.32, ICD-9-D-070.33, ICD-9-D-070.44, ICD-9-D-070.54, ICD-9-D-070.6, ICD-9-D-070.9, ICD-9-D-570.x, ICD-9-D-571.x, ICD-9-D-573.3, ICD-9-D-573.4, ICD-9-D-573.8, ICD-9-D-573.9, ICD-9-D-V42.7, ICD-10-D-B18.x, ICD-10-D-K70.0-K70.3, ICD-10-D-K70.9, ICD-10-D-K71.3-K71.5, ICD-10-D-K71.7, ICD-10-D-K73.x, ICD-10-D-K74.x, ICD-10-D-K76.0, ICD-10-D-K76.2-K76.4, ICD-10-D-K76.8, ICD-10-D-K76.9, ICD-10-D-Z94.4

Diabetes without Chronic Complication - ICD-9-D-250.0-250.3, ICD-9-D-250.8, ICD-9-D-250.9, ICD-10-D-E10.0, ICD-10-D-E10.l, ICD-10-D-E10.6, ICD-10-D-E10.8, ICD-10-D-E10.9, ICD-10-D-E11.0, ICD-10-D-E11.1, ICD-10-D-E11.6, ICD-10-D-E11.8, ICD-10-D-E11.9, ICD-10-D-E12.0, ICD-10-D-E12.1, ICD-10-D-E12.6, ICD-10-D-E12.8, ICD-10-D-E12.9, ICD-10-D-E13.0, ICD-10-D-E13.1, ICD-10-D-E13.6, ICD-10-D-E13.8, ICD-10-D-E13.9, ICD-10-D-E14.0, ICD-10-D-E14.1, ICD-10-D-E14.6, ICD-10-D-E14.8, ICD-10-D-E14.9

Diabetes with Chronic Complication - ICD-9-D-250.4-250.7, ICD-10-D-E10.2-E10.5, ICD-10-D-E10.7, ICD-10-D-E11.2-E11.5, ICD-10-D-E11.7, ICD-10-D-E12.2-E12.5, ICD-10-D-E12.7, ICD-10-D-E13.2-E13.5, ICD-10-D-E13.7, ICD-10-D-E14.2-E14.5, ICD-10-D-E14.7

Hemiplegia or Paraplegia - ICD-9-D-334.1, ICD-9-D-342.x, ICD-9-D-343.x, ICD-9-D-344.0-344.6, ICD-9-D-344.9, ICD-10-D-G04.1, ICD-10-D-G11.4, ICD-10-D-G80.1, ICD-10-D-G80.2, ICD-10-D-G81.x, ICD-10-D-G82.x, ICD-10-D-G83.0-G83.4, ICD-10-D-G83.9

Renal Disease - ICD-9-D-403.01, ICD-9-D-403.11, ICD-9-D-403.91, ICD-9-D-404.02, ICD-9-D-404.03, ICD-9-D-404.12, ICD-9-D-404.13, ICD-9-D-404.92, ICD-9-D-404.93, ICD-9-D-582.x, ICD-9-D-583.0-583.7, ICD-9-D-585.x, ICD-9-D-586.x, ICD-9-D-588.0, ICD-9-D-V42.0, ICD-9-D-V45.1, ICD-9-D-V56.x, ICD-10-D-I12.0, ICD-10-D-I13.1, ICD-10-D-N03.2-N03.7, ICD-10-D-N05.2-N05.7, ICD-10-D-N18.x, ICD-10-D-N19.x, ICD-10-D-N25.0, ICD-10-D-Z49.0-Z49.2, ICD-10-D-Z94.0, ICD-10-D-Z99.2

Any Malignancy, including Lymphoma and Leukemia, except Malignant Neoplasm of Skin - ICD-9-D-140.x-172.x, ICD-9-D-174.x-195.8, ICD-9-D-200.x-208.x, ICD-9-D-238.6, ICD-10-D-C00.x-C26.x, ICD-10-D-C30.x-C34.x, ICD-10-D-C37.x-C41.x, ICD-10-D-C43.x, ICD-10-D-C45.x-C58.x, ICD-10-D-C60.x-C76.x, ICD-10-D-C81.x-C85.x, ICD-10-D-C88.x, ICD-10-D-C90-C97.x

Moderate or Severe Liver Disease - ICD-9-D-456.0-456.2, ICD-9-D-572.2-572.8, ICD-10-D-I85.0, ICD-10-D-I85.9, ICD-10-D-I86.4, ICD-10-D-I98.2, ICD-10-D-K70.4, ICD-10-D-K71.1, ICD-10-D-K72.1, ICD-10-D-K72.9, ICD-10-D-K76.5, ICD-10-D-K76.6, ICD-10-D-K76.7

Metastatic Solid Tumor - ICD-9-D-196.x-199.x, ICD-10-D-C77.x-C80.x

AIDS/HIV - ICD-9-D-042.x-044.x, ICD-10-D-B20.x-B22.x, ICD-10-D-B24.x
