# Supplementary material for: Complication, revision, and readmission rates following reverse total shoulder arthroplasty in wheelchair-dependent patients
Source: J Shoulder Elb Arthroplast. 2026 Feb 9;10(1-2):100003. doi: 10.1016/j.jsea.2026.100003 (PMC13012886; doi:10.1016/j.jsea.2026.100003)
Supplement: Supplemental File 2 [file mmc2.docx]

**Supplemental File 2.** ICD-9, ICD-10, and CPT codes for Identification of Outcomes

**Revision:**

ICD-9-P-8197 - Revision of joint replacement of upper extremity

ICD-10-P-0RWJ0JZ - Revision of Synthetic Substitute in Right Shoulder Joint, Open Approach

ICD-10-P-0RWK0JZ - Revision of Synthetic Substitute in Left Shoulder Joint, Open Approach

CPT-23473: Revision of total shoulder arthroplasty for one component, such as the humeral or glenoid component

CPT-23474: Revision of total shoulder arthroplasty for both components

**Mechanical Complications:**

ICD-10-D-T84.018A - Broken internal joint prosthesis, other site

ICD-10-D-T84.019A - Broken internal joint prosthesis, unspecified site

ICD-10-D-T84.038A - Mechanical loosening of other internal prosthetic joint

ICD-10-D-T84.039A - Mechanical loosening of unspecified internal prosthetic joint

ICD-10-D-T84.058A - Periprosthetic osteolysis of other internal prosthetic joint

ICD-10-D-T84.059A - Periprosthetic osteolysis of unspecified internal prosthetic joint

ICD-10-D-T84.068A - Wear of articular bearing surface of other internal prosthetic joint

ICD-10-D-T84.069A - Wear of articular bearing surface of unspecified internal prosthetic joint

ICD-10-D-T84.098A - Other mechanical complication of other internal joint prosthesis

ICD-10-D-T84.099A - Other mechanical complication of unspecified internal joint prosthesis

ICD-9-D-996.41 - Mechanical loosening of unspecified internal prosthetic joint

ICD-9-D-996.43 - Broken internal joint prosthesis

ICD-9-D-996.45 - periprosthetic osteolysis

ICD-9-D-996.46 - Articular bearing surface wear of prosthetic joint

ICD-9-D-996.47 - mechanical complication of an unspecified internal joint prosthesis

ICD-10-D-T84.110A - Breakdown (mechanical) of internal fixation device of right humerus

ICD-10-D-T84.111A - Breakdown (mechanical) of internal fixation device of left humerus

ICD-10-D-T84.112A - Breakdown (mechanical) of internal fixation device of bone of right forearm

ICD-10-D-T84.113A - Breakdown (mechanical) of internal fixation device of bone of left forearm

ICD-10-D-T84.119A - Breakdown (mechanical) of internal fixation device of unspecified bone of limb

ICD-10-D-T84.12 - Displacement of internal fixation device of bones of limb

ICD-10-D-T84.120A - Displacement of internal fixation device of right humerus

ICD-10-D-T84.121A - Displacement of internal fixation device of left humerus

ICD-10-D-T84.122A - Displacement of internal fixation device of bone of right forearm

ICD-10-D-T84.123A - Displacement of internal fixation device of bone of left forearm

ICD-10-D-T84.129A - Displacement of internal fixation device of unspecified bone of limb

ICD-10-D-T84.190A - Other mechanical complication of internal fixation device of right humerus

ICD-10-D-T84.191A - Other mechanical complication of internal fixation device of left humerus

ICD-10-D-T84.192A - Other mechanical complication of internal fixation device of bone of right forearm

ICD-10-D-T84.193A - Other mechanical complication of internal fixation device of bone of left forearm

ICD-10-D-T84.199A - Other mechanical complication of internal fixation device of unspecified bone of limb

ICD-9-D-996.49 - Other mechanical complication of other internal orthopedic device, implant, and graft

ICD-10-D-T84.210A - Breakdown (mechanical) of internal fixation device of bones of hand and fingers

ICD-10-D-T84.218A - Breakdown (mechanical) of internal fixation device of other bones

ICD-10-D-T84.220A - Displacement of internal fixation device of bones of hand and fingers

ICD-10-D-T84.228A - Displacement of internal fixation device of other bones

ICD-10-D-T84.290A - Other mechanical complication of internal fixation device of bones of hand and fingers

ICD-10-D-T84.298A - Other mechanical complication of internal fixation device of other bones

ICD-10-D-T84.318A - Breakdown (mechanical) of other bone devices, implants and grafts

ICD-10-D-T84.32 - Displacement of other bone devices, implants and grafts

ICD-10-D-T84.320A - Displacement of electronic bone stimulator

ICD-10-D-T84.328A - Displacement of other bone devices, implants and grafts

ICD-10-D-T84.390A - Other mechanical complication of electronic bone stimulator

ICD-10-D-T84.398A - Other mechanical complication of other bone devices, implants and grafts

ICD-10-D-T84.410A - Breakdown (mechanical) of muscle and tendon graft

ICD-10-D-T84.418A - Breakdown (mechanical) of other internal orthopedic devices, implants and grafts

ICD-10-D-T84.420A - Displacement of muscle and tendon graft

ICD-10-D-T84.428A - Displacement of other internal orthopedic devices, implants and grafts

ICD-10-D-T84.490A - Other mechanical complication of muscle and tendon graft

ICD-10-D-T84.498A - Other mechanical complication of other internal orthopedic devices, implants and grafts

**Dislocation:**

ICD-10-D-T84.028A - Dislocation of other internal joint prosthesis

ICD-10-D-T84.029A - Dislocation of unspecified internal joint prosthesis

ICD-9-D- 996.42 - Dislocation of prosthetic joint

**Breakage:**

ICD-10-D-T84.018A - Broken internal joint prosthesis, other site

ICD-10-D-T84.019A - Broken internal joint prosthesis, unspecified site

ICD-10-D-T84.110A - Breakdown (mechanical) of internal fixation device of right humerus

ICD-10-D-T84.111A - Breakdown (mechanical) of internal fixation device of left humerus

ICD-10-D-T84.112A - Breakdown (mechanical) of internal fixation device of bone of right forearm

ICD-10-D-T84.113A - Breakdown (mechanical) of internal fixation device of bone of left forearm

ICD-10-D-T84.119A - Breakdown (mechanical) of internal fixation device of unspecified bone of limb

ICD-10-D-T84.210A - Breakdown (mechanical) of internal fixation device of bones of hand and fingers

ICD-10-D-T84.218A - Breakdown (mechanical) of internal fixation device of other bones

ICD-10-D-T84.318A - Breakdown (mechanical) of other bone devices, implants and grafts

ICD-10-D-T84.410A - Breakdown (mechanical) of muscle and tendon graft

ICD-10-D-T84.418A - Breakdown (mechanical) of other internal orthopedic devices, implants and grafts

ICD-9-D-996.43 - Broken prosthetic joint implant

ICD-9-D-996.49 - Other mechanical complication of other internal orthopedic device, implant, and graft

**Loosening:**

ICD-10-D-T84.038A - Mechanical loosening of other internal prosthetic joint

ICD-10-D-T84.039A - Mechanical loosening of unspecified internal prosthetic joint

ICD-9-D-996.41 - Mechanical loosening of prosthetic joint

**Infection:**

ICD-10-D-T84.50XA - Infection and inflammatory reaction due to unspecified internal joint prosthesis

ICD-10-D-T84.59XA - Infection and inflammatory reaction due to other internal joint prosthesis

ICD-10-D-T84.60XA - Infection and inflammatory reaction due to internal fixation device of unspecified site

ICD-10-D-T84.610A - Infection and inflammatory reaction due to internal fixation device of right humerus

ICD-10-D-T84.611A - Infection and inflammatory reaction due to internal fixation device of left humerus

ICD-10-D-T84.612A - Infection and inflammatory reaction due to internal fixation device of right radius

ICD-10-D-T84.613A - Infection and inflammatory reaction due to internal fixation device of left radius

ICD-10-D-T84.614A - Infection and inflammatory reaction due to internal fixation device of right ulna

ICD-10-D-T84.615A - Infection and inflammatory reaction due to internal fixation device of left ulna

ICD-10-D-T84.619A - Infection and inflammatory reaction due to internal fixation device of unspecified bone of arm

ICD-10-D-T84.69XA - Infection and inflammatory reaction due to internal fixation device of other site

ICD-10-D-T84.7XXA - Infection and inflammatory reaction due to other internal orthopedic prosthetic devices, implants and grafts

ICD-9-D-996.66 - Infection and inflammatory reaction due to internal joint prosthesis

ICD-9-D-996.67 - Infection and inflammatory reaction due to other internal orthopedic device, implant, and graft

**Other complications of internal orthopedic prosthetic devices, implants, grafts:**

ICD-10-D-T84.8 - Other specified complications of internal orthopedic prosthetic devices, implants and grafts

ICD-10-D-T84.81XA - Embolism due to internal orthopedic prosthetic devices, implants and grafts

ICD-10-D-T84.82XA - Fibrosis due to internal orthopedic prosthetic devices, implants and grafts

ICD-10-D-T84.83XA - Hemorrhage due to internal orthopedic prosthetic devices, implants and grafts

ICD-10-D-T84.84XA - Pain due to internal orthopedic prosthetic devices, implants and grafts

ICD-10-D-T84.85XA - Stenosis due to internal orthopedic prosthetic devices, implants and grafts

ICD-10-D-T84.86XA - Thrombosis due to internal orthopedic prosthetic devices, implants and grafts

ICD-10-D-T84.89XA - Other specified complication of internal orthopedic prosthetic devices, implants and grafts

ICD-9-D-996.77 - Other complications due to internal joint prosthesis

ICD-9-D-996.78 - Other complications due to other internal orthopedic device, implant, and graft

**Unspecified complication of internal orthopedic prosthetic device, implant and graft:**

ICD-10-D-T84.9XXA - Unspecified complication of internal orthopedic prosthetic device, implant and graft

ICD-9-D-996.77 - Other complications due to internal joint prosthesis

ICD-9-D-996.78 - Other complications due to other internal orthopedic device, implant, and graft

**Other intraoperative and postprocedural complications and disorders of the musculoskeletal system:**

ICD-10-D-M96.89 - Other intraoperative and postprocedural complications and disorders of the musculoskeletal system

ICD-9-D-99799 - Complications affecting other specified body systems, not elsewhere classified

**Periprosthetic Fracture:**

ICD-10-D-M97.31XD - Periprosthetic fracture around internal prosthetic right shoulder joint

ICD-10-D-M97.32XA - Periprosthetic fracture around internal prosthetic left shoulder joint

ICD-9-D-996.44 - Peri-prosthetic fracture around prosthetic joint

**Surgical operation with implant of artificial internal device as cause of abnormal reaction/complication:**

ICD-10-D-Y83.1 - Surgical operation with implant of artificial internal device as the cause of abnormal reaction of the patient, or of later complication, without mention of misadventure at the time of the procedure

ICD-9-D-E8781 - Surgical operation with implant of artificial internal device causing abnormal patient reaction, or later complication, without mention of misadventure at time of operation

**Flail Joint:**

ICD-10-D-M25.21 - Flail joint, shoulder

ICD-10-D-M25.211 - Flail joint, right shoulder

ICD-10-D-M25.212 - Flail joint, left shoulder

ICD-10-D-M25.219 - Flail joint, unspecified shoulder

ICD-9-D-Other joint derangement, not elsewhere classified, shoulder region
